# Factors influencing mask use and physical distancing for COVID-19: A qualitative evidence synthesis

**DOI:** 10.4102/jphia.v16i2.614

**Published:** 2025-04-14

**Authors:** Dachi I. Arikpo, Okwu A. Onyema, Afiong O. Oku, Chibueze Meremikwu, Hannah Hamilton-Hurwitz, João P. Toledo, Kathleen Dunn, April Baller, Helen J. Smith, Martin M. Meremikwu

**Affiliations:** 1 Cochrane Nigeria, Institute of Tropical Diseases Research and Prevention, University of Calabar Teaching Hospital, Calabar, Nigeria; 2 Department of Sociology, University of Calabar, Calabar, Nigeria; 3 Department of Community Medicine, University of Calabar Teaching Hospital, Calabar, Nigeria; 4 Department of Mass Communication, Hezekiah University, Umudi, Imo state, Nigeria; 5 World Health Organization, Geneva, Switzerland; 6 Public Health Agency of Canada, Ottawa, Canada; 7 International Health Consulting Services Ltd, Liverpool, United Kingdom; 8 Department of Pediatrics, University of Calabar Teaching Hospital, Calabar, Nigeria

**Keywords:** COVID-19, SARS-CoV-2, infection prevention and control, face masks, masks, physical distancing, PPE, qualitative evidence synthesis, systematic review

## Abstract

**Background:**

The World Health Organization (WHO) recommends a bundle of precautions to reduce community transmission of COVID-19, including mask use and physical distancing. However, there is evidence that suggests poor adherence to these health measures community settings.

**Aim:**

To summarise qualitative research evidence on the perceptions and factors influencing masks use and physical distancing in the context of the COVID-19 pandemic.

**Setting:**

We included studies conducted in community settings.

**Method:**

An electronic database search was conducted using search terms derived from the inclusion criteria and combined in a peer-reviewed search strategy. Thirty studies were sampled. Qualitative data analysis was performed using the thematic synthesis approach. The confidence in each review finding was ascertained using the Grading of Recommendations, Assessment, Development and Evaluations – Confidence in the evidence from Reviews of Qualitative Research (GRADE-CERQual) approach.

**Results:**

Ten analytical themes of low to high confidence were identified. Values, belief systems and cultural norms shaped the perception and uptake of mask use and physical distancing. Key barriers included the cost of masks, limited infrastructure for spatial separation and inconsistent political or government messaging, while visual cues and social responsibility facilitated adherence.

**Conclusion:**

Personal values and preferences influenced individuals’ adherence to these public health measures. Political or government messaging is important to aid understanding and adherence.

**Contribution:**

Insights provided by this synthesis can support future emergency preparedness and response to outbreaks of acute respiratory infections by providing policy makers with information needed to make contextually relevant recommendations to enhance adherence.

## Introduction

The World Health Organization (WHO) recommends using a bundle of interventions, such as mask wearing and physical distancing to reduce community transmission of severe acute respiratory syndrome coronavirus 2 (SARS-CoV-2).^1^ Implementation and adherence to these public health and social measures (PHSM) can reduce the spread of SARS-CoV-2, especially in community settings.^2^

Masks serve as a source control measure by providing a protective barrier against respiratory infectious particles.^3^ Medical masks are typically available for purchase and use by the general public; however, the unforeseen nature of the COVID-19 outbreak led to acute shortages of masks and the need to improvise by using non-medical masks (i.e., cloth masks).^4,5^ Other public health measures, such as physical distancing, limit close contact between individuals, potentially lowering the risk of transmitting respiratory infections.^1,6^

In response to the COVID-19 pandemic, mask use and physical distancing policies were instituted at global, regional and national levels to contain the spread of the virus. However, poor adherence to this public health advice, especially in non-healthcare settings, was observed and reported.^7,8^ Therefore, it is critical to investigate the reasons and factors influencing non-implementation in order to strengthen policies and their implementation.

Ill adherence with mask use and physical distancing policies in the context of COVID-19 may stem from varying perceptions of the effectiveness, acceptability and feasibility of these PHSM and the lived experiences of community members following their implementation. Synthesis of evidence from qualitative research is important to broaden our understanding of contextual factors that may influence uptake of mask use and physical distancing across different settings. The WHO also uses a robust methodology for guideline development, which includes using qualitative data to inform relevant domains of the Evidence-to-Decision framework.^9^ This review was commissioned by WHO to bridge the knowledge gap in the evidence base for the 2023 update of the living guidelines on infection prevention and control in the context of COVID-19.^1^ The database search performed at the time of conducting this study showed no existing QES on perceptions and factors influencing uptake of mask and physical distancing in community settings in the context of COVID-19. It was also necessary to synthesise the documented lived experiences, perceptions and challenges affecting the implementation and uptake of these PHSM to inform future emergency preparedness.

### Aim

This qualitative evidence synthesis (QES) aimed to (1) synthesise the perceptions and experiences of the general public on the use of masks and physical distancing in community settings in the context of COVID-19 and (2) identify the contexts and conditions that influence uptake of these infection prevention and control (IPC) measures.

## Methods

### Study design

Qualitative evidence synthesis was performed using the methodology prescribed by Cochrane,^10,11,12^ and reported according to the stipulations of the ‘Enhancing transparency in reporting the synthesis of qualitative research’ (ENTREQ) and Preferred Reporting Items for Systematic Reviews and Meta-Analyses (PRISMA) checklists.^13,14^ The review protocol was registered on the International Prospective Register of Systematic Reviews (PROSPERO, CRD42022356383).

### Inclusion criteria

The SPICE (setting, perspective, phenomenon of interest, comparison and evaluation) framework,^15^ was used to determine the inclusion criteria (Table 1). The literature search was targeted at primary studies published in response to the COVID-19 pandemic to inform the update of the WHO COVID-19 IPC guidelines. Only relevant studies published from 01 January 2020 to the date of the search (05 September 2022) were considered for inclusion in the QES.

**TABLE 1:** Inclusion criteria (SPICE framework).

Concept	Description
Setting	Healthcare facilities, including care homes. Community including households. In any geographical location and level of healthcare.
Perspective (population)	Stakeholders: Healthcare workers involved in patient care and those not involved in patient care. Healthcare policymakers. Health facility clients (including residents of care homes, recipients of care – inpatients and outpatients) and visitors. Community members – general public and members of households.
Phenomenon of interest	Physical barriers and distancing interventions for COVID-19 infection prevention and control.
Intervention	Physical barriers and distancing for infection prevention, including: PPEs (e.g., mask, coveralls, gowns, shoe covers, N95 respirators, gloves, goggles, face shields). Physical distancing. Engineering controls (air cleaning and purifier technologies; spatial separation using physical barriers).
Evaluation (*outcomes*)	Perceptions of stakeholders, including views, attitudes, experiences, perspectives. Factors influencing uptake (barriers and facilitators) at the individual, provider, health system, community and social-political levels.
Study design	Primary studies conducted using qualitative study designs, including ethnography, phenomenology, case studies, grounded theory studies, applied qualitative research, mixed-methods and process evaluations. Studies using both qualitative methods for data collection (e.g. observations, focus group discussions, individual interviews) and qualitative methods for data analysis (e.g., thematic analysis, framework analysis, content analysis and grounded theory).
Date limits	01 January 2020 to 05 September 2022; to capture research published in response to the COVID-19 outbreak.

PPE, personal protective equipment.

### Search strategy

Information scientists from Cochrane (G.C., M.C., E.C.) collaborated to design and implement the peer-reviewed search strategy (Appendix 1) on the topic of interest in health and care, and community settings. The electronic search was performed on one database – MEDLINE (Ovid). In addition, the reviewers searched the reference list of related full-text publications to identify any additional studies for inclusion.

### Study selection and sampling

Records retrieved by the search were uploaded to the Endnote Reference Management software.^16^ The records were deduplicated, and the eligibility criteria were applied to the remaining records. Working in pairs, one author (either C.M. or O.A.O) screened all titles, abstracts and full texts of potentially eligible studies using a pre-piloted eligibility screening form, while a second author (either D.I.A. or A.O.O.) validated screening output. Inconsistencies in the selection process were resolved by consulting a third review author (H.J.S.).

Through full-text screening, 78 studies were included in the review; the authors considered this number too many to analyse efficiently. Therefore, maximum variation purposive sampling was applied to select studies for the synthesis.^17,18^ A three-step sampling frame with the following parameters ‘data richness’, ‘closeness of the study to our synthesis objective’ and ‘geographical spread or representation’ was developed and used. Data richness was ascertained using the data richness scale by Ames and colleagues.^19^ Prior to applying the sampling criteria, each included study was categorised into intervention groups (PPE and masks in healthcare settings, masks in the community and physical distancing in healthcare and community settings). Physical distancing was not restricted to maintaining a distance of 1 m or 2 m but was a general concept of maintaining space between persons. Excluded studies, studies not sampled and the reasons for excluding or non-sampling were duly documented.

### Data extraction

Characteristics and outcomes reported by the sampled studies were inputted into a pre-piloted data extraction spreadsheet in Microsoft^®^ Excel. Themes and supporting quotes were then extracted into two other spreadsheets. One review author (O.A.O) extracted data from the sampled studies, and two authors (D.I.A., A.O.O.) verified all extracted data for accuracy and completeness. Discrepancies were corrected by the verifying authors or by consulting the wider author team.

### Assessment of methodological limitations of sampled studies

An adapted version of the Critical Appraisal Skills Programme (CASP) tool for qualitative studies was used to assess the methodological limitations of the sampled studies.^20^ The tool evaluates the appropriateness or adequacy of descriptions of the study context, sampling strategy, data collection and analysis, evidence supporting the findings, reflexivity and ethical considerations. Two authors (O.A.O and D.I.A.) appraised the studies independently and reached a consensus on the final judgement for each domain. The quality assessment was not an eligibility criterion for including studies in this QES.

### Data analysis

Two review authors (D.I.A., O.A.O) performed the initial coding of extracts independently. They identified discrepancies in coding and reached a consensus on the coding approach. Subsequently, one author (D.I.A.) coded extracted texts from the sampled studies line by line while a second author (H.J.S.) verified the coding. Codes were iteratively identified, developed and reviewed in accordance with the extracted text’s contents and meaning.

A thematic synthesis for perception and experiences of mask use and physical distancing was performed.^21^ This synthesis approach is highly recommended by Cochrane and allowed for descriptive themes to be induced directly from the data and organised using ‘constant comparison’ methods.^22^ Analytical themes were subsequently developed by re-grouping descriptive themes. The thematic synthesis method was well-suited for delving into the viewpoints and encounters of stakeholders, and adequately supports the development of descriptive and analytical themes.^23,24^

Data concerning the factors affecting uptake of mask and physical distancing were synthesised through a deductive approach, aligning descriptive themes with a pre-existing framework. The Supporting the Use of Research Evidence (SURE) framework,^25^ was deemed the best-fit framework for this synthesis. The SURE framework was selected because it identifies factors that influence the implementation of a policy option at the level of the individual, and the social and political context. Data were extracted against the SURE framework domains and, from these, developed descriptive and then analytical themes. The hybrid approach of the synthesis, coding process and development of descriptive and analytical themes was discussed and agreed to by all authors.

### Assessing the confidence in the review findings

Use of GRADE-CERQual determined the confidence level (high, moderate, low or very low) for each review finding (analytical theme).^26^ One review author (D.I.A.) evaluated the confidence of each finding across four domains. A second author (H.J.S.) verified the judgements and ratings. The overall assessment was determined by the author team’s consensus.

### Review author reflexivity

The author team is a multidisciplinary team of researchers working in evidence-based healthcare. They include academics and clinicians with backgrounds in social sciences, public health, nursing and medicine, and have experience in conducting scientific research, including qualitative research, synthesis of qualitative research and epidemiology. The majority of the author team also have considerable knowledge of existing social, behavioural conceptual frameworks that explain health behaviour. As a review team, we understood that our perspectives and experiences of the COVID-19 pandemic may influence how we collect, analyse and interpret the data. All the authors had experienced the COVID-19 pandemic and generally considered IPC strategies essential for mitigating the spread of the virus, but did not have clear expectations of the review findings. The authors were mindful of their inclinations throughout the conduct of the QES and minimised potential biases in the analysis and interpretation of the review findings using the refutational analysis approach.^27^ We believe the multidisciplinary nature of the team allowed for rich insights and balanced views on the findings and interpretation of the evidence. All synthesis findings were discussed and agreed on as a team.

### Ethical considerations

This article followed all ethical standards for research without direct contact with human or animal subjects.

## Results

### Included studies

**FIGURE 1:**
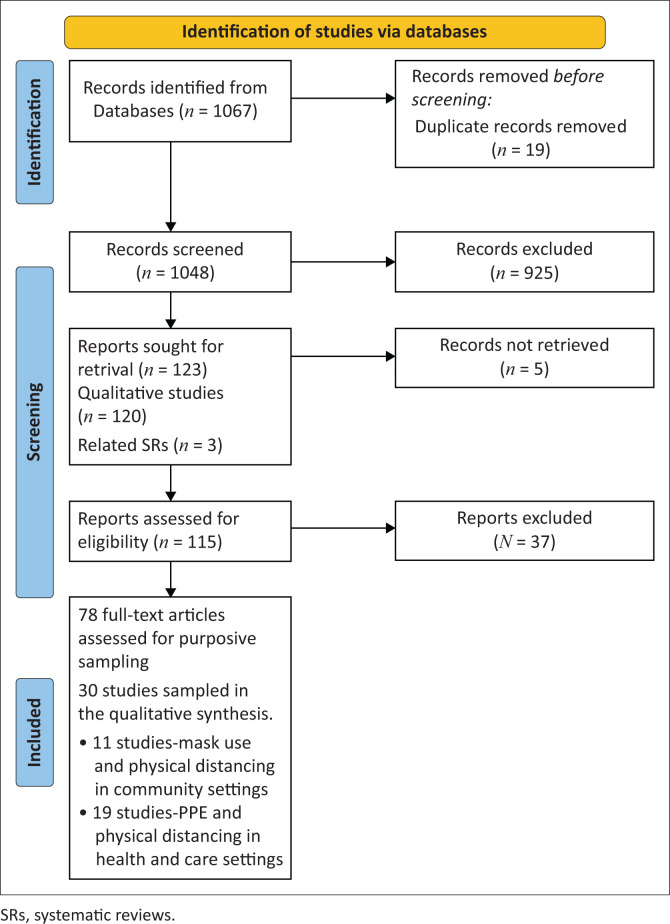
Preferred Reporting Items for Systematic Reviews and Meta- Analyses (PRISMA) flow diagram.

The database search returned 1067 records, including studies conducted in both community and health and care settings. Following the screening of titles and abstracts, full-text and sampling, 30 studies were included and synthesised for both settings. Post hoc, we realised it would be more efficient to present the synthesis results for health and care settings, and community settings in separate reports. In this review, we present the results of the synthesis of 11 studies on mask use and physical distancing in community settings.^28,29,30,31,32,33,34,35,36,37,38^ The perceptions and experiences of personal protective equipment (PPE) use and physical distancing in healthcare settings (*n* = 19) are reported in a separate synthesis.^39^ The Preferred Reporting Items for Systematic Reviews and Meta-Analyses (PRISMA) flow diagram (Figure 1) details the study selection process.

Following the World Bank classifications, four of the sampled studies were set in three low-income countries (LICs): Ethiopia,^29,32^ Mali^36^ and the Democratic Republic of the Congo,^37^ (Table 2). Another study^30^ was set in a lower-middle-income country (LMIC) – Tanzania. One study,^38^ was from an upper-middle-income country (UMIC) – South Africa. Four studies were from three high-income countries (HICs): the United States (US),^31^ United Kingdom (UK)^33,35^ and Ireland.^34^ One of the studies was a multi-country study (including participants from 20 European countries and two Asian countries) conducted across different income settings.^28^

**TABLE 2:** Characteristics of sampled studies.

Study ID	Study setting	Aim	Study design	Perspective	Participants	Sample size	Data-collection method	Context	Method of data analysis	Overall assessment of methodological limitations
**Masks (*n* = 4)**										
Martinelli^28^ (20 European countries, 2 Asian countries)	Community	To identify the diversity of sociocultural, ethical and political meanings attributed to masks, how they might impact public health policies and how they should be considered in health communication	Primary qualitative study	Community members	Researchers	29	Online survey using open-ended questionnaires	Urban	Thematic analysis	Moderate
Tsegaye^29^ (Ethiopia)	Community	Examine factors influencing COVID-19-related behavioural decisions in the Nguenyyiel refugee camp located in Gambella, Ethiopia	Primary qualitative study	Community members - Persons living in IDP or refugee camps	Elderly men, elderly women, middle-aged men, middle-aged women, male youth, female youth, members of the refugee central committee, community leaders andhumanitarian organisation staff members	47	Focus group discussions and key informant interviews	Rural	Thematic analysis	Minor
Mohamed^30^ (Tanzania)	Community	To assess access to WASH and other preventive measures against COVID-19 among people with disabilities	Primary qualitative study	Community members	People with disabilities	102	Focus group discussions and key informant interviews	Mixed	Content analysis	Minor
Scheid^31^ (US)	Community	To use a mixed-methods approach to examine if facemask use impacts the perception of exertion during MVPA	Mixed-methods study	Community members	Physically active adults	72	Online survey using open-ended questionnaires	Urban	Thematic analysis	Minor
**Physical distancing (*n* = 7)**										
Hailu^32^ (Ethiopia)	Community	To assess the compliance, barriers, and facilitators to social distancing measures for the prevention of COVID-19 in Northwest Ethiopia	Mixed-methods study	Community members	Religious leaders, community health workers, traffic police, drivers and community leaders	12	Interviews	Urban	Thematic analysis	Moderate
Berry^33^ (UK)	Community	Explores the facilitators of and barriers to social distancing for young people during the COVID-19 pandemic	Primary qualitative study	Community members	Young people	477	Online survey using open-ended questionnaires	Urban	Reflexive thematic analysis from a subtle realist approach	Minor
Farrell^34^ (Ireland)	Community	To qualitatively explore barriers and facilitators of physical distancing in the context of COVID-19 pandemic	Primary qualitative study	Health facility clients	Individuals living in the Republic of Ireland between September 2020 and October 2020	25	Interviews	Urban	Reflexive thematic analysis	Minor
Eraso^35^ (UK)	Community	To analyse non-adherence to a cluster of social distancing rules	Mixed-methods study	Community members	Individuals (North London residents)	30	Interviews	Urban	Thematic content analysis	Minor
Ag-Ahmed^36^ (Mali)	Community	To contribute to an effort to contextualise and adapt physical distancing measures targeting COVID-19 among IDPs in Mali	Primary qualitative study	Community members	IDPs and camp managers	68	Interviews and focus group discussions	Urban	Thematic content analysis	Minor
Claude^37^ (Democratic Republic of the Congo)	Community	To contribute to the improvement of prevention strategies against COVID-19 in IDP camps in the DRC	Mixed-methods study	Community members	IDPs	23	Focus group discussions	Rural	Thematic analysis	Minor
Nyanshanu^38^ (South Africa)	Community	To explore the impact of lockdown during COVID-19 among people living in informal settlements	Primary qualitative study	Community members	People living in informal settlements	30	Interviews	Urban	Interpretive phenomenological analysis	Moderate

Note: Please see the full reference list of the article Arikpo DI, Onyema AO, Oku AO, et al. Factors influencing mask use and physical distancing for COVID-19: A qualitative evidence synthesis. J Public Health Africa. 2025;16(2), a614. https://doi.org/10.4102/jphia.v16i2.614, for more information.

COVID-19,coronavirus disease 2019; IDP, internally displaced person; WASH, water, sanitation and hygiene; MVPA, moderate to vigorous physical activity; DRC, Democratic Republic of the Congo.

Four of the sampled studies reported perceptions and experiences of mask use,^28,29,30,31^ and seven studies reported the perception of physical distancing.^32,33,34,35,36,37,38^ All sampled studies were primary qualitative studies except four studies that were mixed-method studies.^31,32,35,37^ Study participants were individual community members, young people, people with disabilities, physically active adults, people living in informal settlements internally displaced persons (IDPs), IDP camp managers, refugees, members of the refugee central committee, community leaders, religious leaders, community health workers, traffic police, drivers, humanitarian organisation staff members and researchers (Table 2).

The methodological quality of the studies ranged from minor methodological limitations (*n* = 8 studies) to moderate limitations (*n* = 3 studies) (Table 2). Most studies provided descriptive information on the study context, sampling strategy, data collection and analysis approaches and ethical considerations. They also offered underlying data to support their findings. Only two studies explicitly reported on researcher reflexivity.^33,34^ The QES authors discussed the implications of the absence of reflexivity on each finding supported by studies that did not report on researcher reflexivity.

### Review findings

Ten analytical themes emerged from the synthesis: six related to community members’ perception and experience of mask use and four on physical distancing (Table 3). The analytical themes, corresponding descriptive themes and sample quotes are also presented in Table 3. The outcome of the GRADE-CERQual assessment is presented with each review finding and details are shown in the Summary of Qualitative Findings Table (Table 4). Four findings were rated as high confidence, four as moderate confidence and two as low confidence. The key reasons for downgrading the certainty of evidence were methodological limitations and data adequacy.

**TABLE 3:** Synthesis results (themes and supporting quotes) – masks use and physical distancing in community settings.

S/N	Analytical theme	Descriptive themes (review findings)	Studies contributing to the review finding	Supporting data (example quote)
1	Belief systems discourage mask wearing	Culture, beliefs and norms	Tsegaye^29^ (Ethiopia);	‘Some participants recognised that *a certain sense of fatalism, inherent in Nuer culture*, may also have hindered, to some extent, the uptake of preventive measures in the camp. The Nuer considered death to be a normal thing that should not be feared. *This means that people are not too afraid of dying of COVID-19 and are therefore not very likely to adopt preventive* measures.’ (Tsegaye, 2021, Ethiopia) [*italics added by author*]
		Perceived low severity and risk of COVID-19	Tsegaye^29^ (Ethiopia)	‘The *community does not believe that the disease is serious* enough to kill people. They *ignore the disease considering it not that serious*’. (Tsegaye, 2021, Ethiopia) [*italics added by author*]
2	Safety but false security in mask wearing	False sense of security with mask wearing	Martinelli^28^ (20 European countries, China, South Korea)	‘I believe *the benefits of face masks may be overestimated and lead us into a false sense of security* in which we take unwarranted risks – such as touching more objects and neglecting handwashing or going outside when suffering from a cough or cold. Therefore, *my preference would be to give greater attention to other steps such as providing screens and visors for workers in public facing roles and reinforcing protective mechanisms around social distancing*.’ (Martinelli, 2020, multiple countries) [*italics added by author*]
3	Masks prevent effective communication and affect relationships	Masks affect social relations	Martinelli^28^ (20 European countries, China, South Korea)	‘The use of a mask is seen as an act of responsibility and altruism. However, I notice that *people with masks tend to avoid personal interaction and to decrease the time they talk to each other.* They avoid looking at others.’ (Martinelli, 2020, multiple countries) [*italics added by author*]
4	Coping with mask wearing during physical activity	Masks boosted physical endurance	Scheid^31^ (US)	‘I definitely *believed it boosted my endurance levels* by a lot.’ Participant X … describing *feeling stronger* compared to the pre-mask mandate. The participant attributed this feeling to a ‘placebo or it could be a hypoxic effect” due to mask wearing …’ (Scheid^31^, US) [*italics added by author*]
		Mask wearing didn’t affect physical activity routines	Scheid^31^ (US)	‘…These participants *described the masks as tolerable and expressed that there were no changes to their routines’*. Participant X reports, ‘working out with a mask on *was uncomfortable, but not enough to keep me from doing it.*’ Participant X described it as, ‘more of an inconvenience than effecting my workout.’ (Scheid^31^, US) [*italics added by author*]
		Discomfort when wearing a mask for physical activity	Scheid^31^ (US)	‘… participants that expressed discomfort, *including sweat, increased temperature, light-headedness, and increased anxiety’*. Participant X described the “mask being *soaked in sweat*”, and Participant X reported, “the mask was *irritating to wear*”. Participants X, X, X described the mask as feeling *hot and uncomfortable*. Participants X described *feeling light-headed while wearing masks while exercising*, and Participant X explained that he/she “*felt more anxious*” due to the mask.’ (Scheid^31^, US) [*italics added by author*]
5	Cost of masks is a barrier for some	Affordability of masks	Martinelli^28^ (20 Euro countries, China, South Korea); Tsegaye, 2021 (Ethiopia), Mohamed 2021 (Tanzania)	‘I could not buy face mask because *I could not afford them.* And sometimes I had very little amount of money, *so I prioritized food over the face mask*’ (a woman with physical disability, FGD Dodoma City Council). (Mohamed 2021, Tanzania) [*italics added by author*] ‘How many people are there in your home? You mean all your family can wear the same mask? It *may be only you who can afford to buy a mask. Some people cannot afford to buy masks for all family members*.’ (Tsegaye, 2021, Ethiopia) [*italics added by author*]
6	Social and political aspects of mask wearing	The politics of mask wearing	Martinelli^28^ (20 Euro countries, China, South Korea);	‘The *public statement made by wearing* (*or not wearing*) *the face mask did not only address the political standpoints* but have also been used to communicate various societally relevant statements, i.e., stating ethnical, religious, or cultural affiliations. For instance, *many countries that before COVID-19 banned face coverings in public spaces are now mandating it, supporting the idea that the past bans were motivated on the basis of religious/cultural beliefs.’* ‘Ethical and moral dilemmas have already risen, especially in countries where Muslim minorities live. If you ban a burka covering the face due to security reasons, how would you deal with massive usage of face masks?’ (Martinelli, 2020, multiple countries) [*italics added by author*]
		Social responsibility and mask wearing	Martinelli^28^ (20 Euro countries, China, South Korea)	‘For me, unlike other measures to contain the spread of the virus, the *wearing of masks is predominantly a symbol of social cohesion and complying with the rules and not so much a measure to effectively protect myself and others from infection. The few times I saw someone without a mask entering a supermarket or the metro, my first thoughts were about social deviance and the arrogance of ignoring a commonly agreed-upon practice*, and not about the risk of infection.’ (Martinelli, 2020, multiple countries) [*italics added by author*]
		Mandates and compliance with mask wearing	Martinelli^28^ (20 Euro countries, China, South Korea)	‘Also, one can *observe many cases of half-compliance or sham compliance*. For instance, people do wear masks, but slide them down onto their chins or take them off completely while talking to someone on the street or speaking on the phone. And *this is all a performance, keeping their masks somewhere within reach in case of the sudden emergence of police officers*, who are indeed issuing fines for not wearing a mask.’ (Martinelli, 2020, multiple countries) [*italics added by author*]
7	Beliefs and cultural norms compete with the logic of physical distancing	Changing perception of susceptibility and risk	Farrell^34^ (Ireland); Berry^33^ (UK)	‘I think *at the beginning, very beginning I was probably adhering to it a bit more* (*physical distancing*), in that I was really making sure to stand well back. I think *now I’ve been doing less than two metres, definitely*.’ (Farrrell, 2021, Ireland) [*italics added by author*]
		Community distrust of health professionals and disbelief in COVID-19	Berry^33^ (UK)	‘The fact that none of my friends or I have ever caught it despite not social distancing. Can be hard to appreciate its importance.’ (Berry^33^, UK)
		People value cultural norms and beliefs that preclude physical distancing	Ag-Ahmed^36^ (Mali); Berry^33^ (UK); Hailu^32^ (Ethiopia); Farrell^34^ (Ireland)	‘*In the mosque, it is not easy to physically distance* because even if you were trying to, *someone could come and stand right next to you and you can’t just chase them*, especially when there is no more room in the mosque. Then, *we are also told that in a mosque, it is good to stand next to each other when praying, that’s when our prayers are better received* by God.’ (Ag-Ahmed^36^, Mail) [*italics added by author*]
		Security is a greater priority than physical distancing	Claude^37^ (DRC)	‘*If security returns, we will protect ourselves against corona*, we will respect all the measures, and *it’s only at that time that you can start talking about a vaccine or physical distancing* …’ (Claude^37^; DRC) [*italics added by author*]
8	Solidarity in not physically distancing	Peer and social pressure to comply (or not) with physical distancing	Berry^33^ (UK); Farrell^34^ (Ireland); Eraso^35^ (UK)	‘… there’s kind of two, maybe three [friends] that I’d meet up with on a regular basis, and I do find social distancing with them quite difficult, because I always worry like if I said ‘oh can you keep your two metres’ *that there just going to kind of laugh at me and be like what are you doing that for, don’t be ridiculous*.’ (Farrell^34^) [*italics added by author*]
		Difficulty complying with physical distancing among friends and family	Berry^33^ (UK)	‘Limiting how much time I actually spend with people outside my household. The more time you spend with people *it’s easy to become used to it and forget about distancing*.’ (Berry^33^; UK) [*italics added by author*]
9	Physical infrastructure prevents physical distancing	Infrastructure in IDP camps and informal settlements influences physical distancing	Ag-Ahmed^36^ (Mali); Claude^37^ (DRC); Nyanshanu^38^ (South Africa)	‘Here, *it’s not possible ‘ku achana metre moya moya’* [*to stay 1 meter apart; to practice physical distancing*]. If it comes here, we will all die. You have seen the conditions we live in. *Our room measures 6 m by 5 m, and there are 5 families inside*.’ (Claude^37^, DRC) [*italics added by author*]
10	Social and political factors and physical distancing	Social space supports or discourages physical distancing	Farrell^34^ (Ireland); Berry^33^ (UK)	‘… young people felt that *environmental cues and reminders and physical prompts facilitated their ability to keep a distance* from others and reminded them to keep a distance from others … *stickers on the ground, spaced out tables when eating, reminders on social media* /billboards etc.’ (Berry^33^, UK) [*italics added by author*]
		Confusing and contradictory messaging from government or regulatory agencies	Farrell^34^ (Ireland); Berry^33^ (UK); Eraso^35^ (UK); Hailu^32^ (Ethiopia)	‘What I couldn’t understand was at level three, you couldn’t meet anybody in the garden of your own house. But yet you could meet 15, was it 14/15 people outside in a restaurant, or outside in a pub. So, for me, that was crazy. I’m like, more than likely meeting people in your own garden, you’re only meeting a few you might not meet 15. *But I honestly couldn’t understand the logic behind that*,’ (Female, 67, retired). (Farrell^34^; Ireland) [*italics added by author*] ‘*The messaging became very muddle*, and almost a joke (“staying alert”). Friends are not sure of what you can or cannot do. *It was confusing and contradictory*.’ (M11, Eraso^35^) [*italics added by author*] ‘People are not giving much attention to the disease, they are walking in groups and not protecting themselves. On top of this, *the government is not acting uniformly in enforcing the law* (*there is more strict control in houses of worships while it is lenient in other places such as markets and public transportation*) …’ (Hailu^32^, Ethiopia) [*italics added by author*]

Note: Please see the full reference list of the article Arikpo DI, Onyema AO, Oku AO, et al. Factors influencing mask use and physical distancing for COVID-19: A qualitative evidence synthesis. J Public Health Africa. 2025;16(2), a614. https://doi.org/10.4102/jphia.v16i2.614, for more information.

DRC, Democratic Republic of the Congo; US, United States; UK, United Kingdom.

**TABLE 4:** Summary of qualitative findings table – masks use and physical distancing in community settings.

S/N	Summary of review finding (analytical theme)	Studies contributing to this review finding	CERQual assessment	Explanation of CERQual assessment
1	Belief systems discourage mask wearing	Tsegaye^29^ (Ethiopia)	Moderate confidence	No to very minor concerns regarding methodological limitations and coherence, minor concerns regarding relevance and moderate concerns regarding adequacy (one qualitative study contributed thick data to this finding)
2	Safety but false security in mask wearing	Martinelli^28^ (Albania, Austria, Bosnia and Herzegovina, Croatia, Czechia, Estonia, Hungary, Italy, Ireland, Norway, Poland, Portugal, Romania, Serbia, Slovenia, Spain, Sweden, Turkey, Ukraine, United Kingdom, China and South Korea)	Low confidence	No to very minor concerns regarding coherence, minor concerns relevance and moderate concerns regarding methodological limitations (data collection and reflexivity) and adequacy (one qualitative study contributing thick data to this finding)
3	Masks prevent effective communication and affect relationships	Martinelli^28^ (20 European countries, China, South Korea)	Low confidence	No to very minor concerns about coherence and adequacy, and moderate concerns regarding methodological limitations (data collection and reflexivity) and relevance
4	Coping with mask wearing during physical activity	Scheid^31^ (US)	Low confidence	No to very minor concerns about coherence and adequacy, and moderate concerns regarding methodological limitations (data collection and reflexivity) and relevance
5	Cost of masks is a barrier for some	Martinelli^28^ (20 Euro countries, China, South Korea); Mohamed, 2021 (Tanzania); Tsegaye, 2021 (Ethiopia)	Moderate confidence	No to very minor concerns regarding coherence and adequacy, minor concerns about relevance, and moderate concerns regarding methodological limitations (data collection and reflexivity)
6	Social and political aspects of mask wearing	Martinelli^28^ (20 Euro countries, China, South Korea)	Moderate confidence	No to very minor concerns regarding coherence and adequacy, minor concerns regarding relevance, and moderate concerns regarding methodological limitations (data collection and reflexivity)
7	Beliefs and cultural norms compete with the logic of physical distancing	Ag-Ahmed^36^ (Mali); Berry^33^ (UK); Hailu^32^ (Ethiopia); Farrell^34^ (Ireland); Claude^37^ (DRC)	High confidence	No to very minor concerns regarding coherence, relevance and adequacy, minor concerns regarding methodological limitations
8	Solidarity in not physically distancing	Berry^33^ (UK); Farrell^34^ (Ireland); Eraso^35^ (UK); Fauk 2022 (Indonesia);	High confidence	No to very minor concerns regarding methodological limitation, coherence and adequacy, minor concerns regarding relevance
9	Physical infrastructure prevents physical distancing	Berry^33^ (UK); Ag-Ahmed^36^ (Mali); Claude^37^ (DRC); Nyanshanu 2020b (South Africa)	High confidence	No to very minor concerns regarding methodological limitations, coherence and adequacy, minor concerns regarding relevance
10	Social and political factors and physical distancing	Farrell^34^ (Ireland); Berry^33^ (UK); Eraso^35^ (UK); Hailu^32^ (Ethiopia);	High confidence	No to very minor concerns regarding coherence, adequacy and relevance, minor concerns regarding methodological limitations

Note: Please see the full reference list of the article Arikpo DI, Onyema AO, Oku AO, et al. Factors influencing mask use and physical distancing for COVID-19: A qualitative evidence synthesis. J Public Health Africa. 2025;16(2), a614. https://doi.org/10.4102/jphia.v16i2.614, for more information.

CERQual, Confidence in the evidence from Reviews of Qualitative Research; DRC, Democratic Republic of the Congo; US, United States; UK, United Kingdom.

#### Mask use by the general public

*Finding 1*: *Belief systems discourage mask wearing* (*moderate confidence*): In one study conducted in Ethiopia (LIC), belief systems enforced by culture, norms and perceived low severity of COVID-19 discouraged the use of masks.^29^ The study described how the Nuer ethnic groups perceived death as a normal occurrence; therefore, they were not afraid of dying from COVID-19. Community members narrated their perceptions on preventive measures, referring to them as unnecessary as ‘death is not a new thing’. A male youth reported on the community’s perception of COVID-19 stating ‘the community does not believe that the disease is serious enough to kill the people’; therefore, community members were indifferent to adopting preventive measures like mask wearing.^29^

*Finding 2*: *Safety but false security in mask wearing* (*low confidence*): One cross-continental study reported that some community members (researchers) felt that the protection provided by masks had been exaggerated and gave a façade for protection from COVID-19 infection.^28^ Therefore, a *false sense of security was associated with mask wearing*. The researchers described how this idea led to individuals ‘letting their guard down’ and not partaking in additional public health measures, like physical distancing. However, researchers narrated how they would rather adopt a package of public health and social measures, including physical distancing, than promote masks as a standalone measure.

*Finding 3*: *Masks affect social relationships* (*moderate confidence*): In one study, community members perceived mask use as a *barrier to social relations*.^28^ They recounted their observations of physical distancing and mask use leading to a sense of estrangement among people, noting how acquaintances or friends tended to withdraw from social interactions when wearing masks.

*Finding 4*: *Coping with mask wearing during physical activity* (*low confidence*): In one study from a HIC, persons who engaged in physical activity while wearing a mask reported varying experiences.^31^ Some persons expressed how they experienced *discomfort* but that the discomfort experienced was not significant enough to deter them from engaging in their *exercise routines*. The discomfort experienced included feeling light-headed, increased anxiety, sweat and the fact that ‘the mask was irritating to wear’. One participant narrated that they coped well and reported they experienced *boosted physical endurance* levels.

*Finding 5*: *The cost of masks is a barrier for some* (*moderate confidence*): The affordability of masks was described as a barrier to adherence to mask mandates across HICs and LICs.^28,29,30^ For some community members, families of large size and persons living with disabilities who were often enthusiastic about wearing masks noted they were expensive and simply *not affordable*. Confronted by the costs associated with purchasing masks, individuals narrated how they prioritised their sustenance over purchasing masks. Some individuals expressed concern over the absence of regulation and price control in the mask market, particularly noting how retail outlets capitalised on the high demand by selling masks at inflated prices, particularly during the initial stages of the pandemic.

*Finding 6*: *Social and political aspects of mask wearing* (*moderate confidence*): One multi-country study described the social and political factors influencing mask use.^28^ The study reported how community members perceived *wearing masks as a social responsibility* borne out of the need to protect themselves and others from being infected with the virus. This perception contributed to the idea that not using masks in line with mandates was social deviance. The study also narrated how community members disagreed with *mask mandates* at a point and did not appreciate the need for continued enforcement when the spread of the virus was personally viewed as ‘reasonably contained’. Community members were also not very receptive when others requested that they *comply with mask wearing*. The use of masks varied depending on the political context, for example, in some Asian countries like China and South Korea, mask use was a routine for protection against pollutants and the prevention of seasonal flu and common cold.^40^ Therefore, adherence was less problematic; however, the acceptance of masks in other geographic settings like the West was obscured by political, religious and ethnic undertones, influencing use.

*Finding 7*: *Beliefs and cultural norms compete with the logic of physical distancing in community settings* (*high confidence*): Community members in HICs and LICs seemed to *value cultural norms and beliefs that impacted personal perceptions of physical distancing and precluded its practice.*^32,33,34,36,37^ These beliefs included not observing physical distancing while praying in the mosque to guarantee the receipt of prayers by God, eating meals together and sharing hugs, especially in settings with strong familial and social ties. A male IDP from Mali described how eating together reinforced bonds. Another individual from the UK also narrated how hugging is a part of the culture; hence, adherence to physical distancing was difficult. A resident of a conflict-affected area in the DRC described how *security was a greater priority than physical distancing*. Adherence with physical distancing guidelines also waned with time because of personal *changes in perceptions of susceptibility and risk, including* individuals questioning why physical distancing was still enforced when transmission rates were perceived as low and ‘the urgency’ had abated. However, individuals with high-risk perceptions tried to protect themselves and others by complying with physical distancing guidance.

*Finding 8*: *Solidarity in not physically distancing* (*high confidence*): Community members in HICs reported that they experienced *difficulty complying with physical distancing among family, friends and colleagues*.^33,34,35^ This was because of *peer and social pressure* and the ‘sense of camaraderie’ in not observing physical distancing while interacting with family or acquaintances. Friends and acquaintances who had ridden in the same vehicle to a location recounted how they found it contradictory to enforce physical distancing among themselves when they were out of the car. Community members also described how the actions of others – neighbours, friends and colleagues – influenced their adherence or non-adherence to physical distancing guidelines. The study participants recounted being uncertain if it is ethical to insist that an individual maintains a distance from you during an interaction.

*Finding 9*: *Physical infrastructure prevents physical distancing* (*high confidence*): IDPs and community members in four studies conducted across LICs, a UMIC and a HIC described how it was *not feasible to maintain physical distancing* in public spaces at the time of the study.^33,36,37,38^ Overpopulated and overcrowded community settings like IDP camps and informal settlements lacked the spatial capacity to maintain a distance between persons. Even if they did, basic amenities in these settings, like toilets, bathrooms and water sources, were shared and not exclusive to households. An IDP expressed how he feared they [IDPs] would ‘all die’ if there was a COVID-19 outbreak in the camp because it would spread easily because of the inability to maintain physical distance, amongst other infrastructural issues.

*Finding 10*: *Social and political factors and physical distancing in community settings* (*high confidence*): Individuals who were willing to maintain the recommended physical distance narrated how its practice was difficult in small areas and public spaces lacking environmental controls like visual cues, Plexi glass divisions and other forms of spatial separation.^32,33,34,35^ Community members (young people) shared the perspective that the physical environment should support adherence with physical distancing. They suggested the use of ‘technological reminders like an app’ to prompt individuals when they are non-compliant, floor stickers, marking tapes and other prompts to adherence. Individuals also reported that they experienced confusion because of *frequently changing and contradictory messages on physical distancing from government and regulatory agencies* and desired unambiguous and more concise guidelines.

## Discussion

### Summary of main results

The finding of this QES shows that masks were less valued and accepted by community members because of cultural beliefs and the perceived low severity of COVID-19. Community members described experiencing: reduced engagement, communication difficulties, changes in interpersonal relationships and poor support for social relations when they wore masks. Cultural beliefs and norms often competed with the logic of physical distancing. The acceptability of physical distancing was poor in community settings as individuals struggled to comply with guidelines when they were among colleagues, friends or family members. Using visual cues and environmental prompts to create space awareness for physical distancing was generally acceptable to all stakeholders.

### Factors influencing the uptake of masks and physical distancing

Several factors influenced community members’ uptake of masks and physical distancing in low-income and high-income settings. Firstly, the costs of masks was a critical barrier; prices of masks were inflated along the supply chain, highlighting the need for price regulation. For instance, the cost of medical masks in Tanzania was reported as 500.00 Tanzania Shilling (~$0.22), and disadvantaged groups (people with disabilities, internally displaced persons and large families) described how they could not afford them.^30^ In another instance, daily sustenance was prioritised over the purchase of masks because of costs. Secondly, the political and social connotations of masks constituted either a barrier or facilitator based on the study setting. Community members who perceived mask wearing as a social responsibility were more compliant. In some Asian countries, masks were routine before the COVID-19 pandemic, so there were fewer challenges to uptake during the pandemic. Alternatively, there was deviance in settings with divergent and strong political standpoints.

Public spaces and infrastructure, especially in IDP camps and informal settlements, did not support spatial separation; therefore, physical distancing was not feasible. Pre-existing public infrastructure and workspaces could not be expanded or remodelled to accommodate physical distancing requirements. Furthermore, health authorities frequently reviewed guidelines on physical distancing at the onset of the pandemic, which caused miscommunication.

### Implications for practice

The sudden emergence of the pandemic necessitated emergency responses worldwide, leading to significant challenges in implementing public health measures. Furthermore, current epidemiology suggests that COVID-19 may be a sustained concern.^41^ Given the experience and perception of mask use and physical distancing highlighted in this review, it is important to explore options for overcoming the barriers and enhancing the facilitators to adhere with public health guidelines. For the implementation of these public health and social measures at the community level, formative research, orientation and interactions with key community members may aid in the identification of these dissenting beliefs.^42^ Given the importance of political, social and government messaging identified in this study, further research may inform health communication and messaging strategies to aid understanding and overcome barriers to adherence with public health measures.

### Overall completeness of the evidence and implications for research

The studies included in this QES span different socio-economic settings and countries. Studies reporting on mask use and physical distancing were from both HICs and LICs. Most of the studies on masks did not specify the type of mask under consideration. Therefore, we are unable to attribute perceptions or experiences to a specific type of mask. The scope of physical distancing in this review was not limited to maintaining a distance of 1 m or 2 m between individuals as recommended by public health guidelines. This review identified the following research gaps: none of the sampled studies assessed whether cost or other forms of resource requirements for physical distancing were an impediment to complying with physical distancing guidelines. We did not find any primary qualitative studies on the perceptions of environmental controls and other forms of physical barriers. Therefore, there is a need for qualitative studies on the subject.

### Limitations of the study

This rapid review was conducted in response to the update for IPC guidelines for COVID-19. In keeping up with the rapid review methodology, we searched only one database – Ovid MEDLINE. The electronic search was also restricted to studies published in English language within a specific time frame and we only analysed a representative sample of studies. These steps may have limited the range of studies we identified, assessed for eligibility and included in this qualitative evidence synthesis. The review team maintained methodological rigour in conducting the review by using standard Cochrane Methods; the review authors believe that the rigour helped reduce the biases that may be associated with studies of this nature. None of the sampled studies assessed the reported perception of cost or other forms of resource requirements for physical distancing. We also did not identify any primary qualitative studies on the perceptions of environmental controls and other forms of physical barriers.

## Conclusion

This QES presents themes encompassing perceptions, barriers and facilitators concerning the use of masks and physical distancing in community settings. Values and belief systems shaped the perception and uptake of masks in community settings and stood out as a barrier to mask use across different geographic groups. The uptake of physical distancing was primarily hindered by the lack of physical infrastructure to aid space awareness and spatial separation. Primary qualitative studies are needed to understand community members’ perceptions of environmental and engineering control interventions in the context of COVID-19. This QES provides insight into the contextual factors that influence the uptake of PHSM. This information can support future emergency preparedness and response to outbreaks of acute respiratory infections; and it can also be used by public health professionals to design context-specific policies that are both relevant and feasible for implementation in their communities.

## APPENDIX 1: MEDLINE search strategy

Date of search: 05 September 2022

1. Air Filters/ or Air Ionisation/ or Ventilation/
2. (HVAC or HEPA or MERV or CADR or HRV or ERV or &quot;Heating ventilation and air conditioning&quot; or &quot;High Efficiency Particulate Air&quot; or &quot;High Efficiency Particulate Arrestance&quot; or &quot;Minimum
  Efficiency Reporting Value&quot; or &quot;Clean Air Delivery Rate&quot; or &quot;Heat Recovery Ventilator&quot; or &quot;Energy
  Recovery Ventilator&quot;).ti,ab,kf.
3. (ventilation adj2 (unit* or system* or technolog* or vents or equipment or device*)).ti,ab,kf.
4. (air adj2 (cleaner* or cleaning or filter* or filtering or filtration or purifier* or purifying or purification or ultraviolet or ultra violet or UV or UVGI or UVC or actinic or ioni?er* or ioni?ing or ioni?ation or ozone or ozoni?er* or ozoni?ation)).ti,ab,kf.
5. (ion generator* or electrostatic precipitator*).mp.
6. or/1-5
7. air pollutants/ or air pollutants, occupational/
8. air pollution/ or air pollution, indoor/
9. Air/ or Air Microbiology/
10. Aerosols/ or Inhalation Exposure/
11. or/7–10
12. filtration/ or Ultraviolet Rays/ or Ultraviolet Therapy/
13. 11 and 12
14. 6 or 13
15. patient isolators/ or protective devices/ or masks/ or personal protective equipment/ or exp protective clothing/ or exp respiratory protective devices/
16. ((protective adj2 (clothing or equipment or device?)) or PPE or glove? or gown?).ti,ab,kf.
17. (mask? or facemask* or &quot;face mask*&quot; or respirator? or N95 or N99 or FFP2 or FFP3 or &quot;Filtering face piece?&quot; or &quot;Filtering facepiece?&quot; or faceshield? or &quot;face shield?&quot; or goggles).ti,ab,kf.
18. or/15–17
19. ((engineering or environmental) adj2 control?).ti,ab,kf.
20. physical distancing/
21. ((physical* or social* or spa?ial) adj2 (barrier? or distanc* or separation or proximity)).ti,ab,kf.
22. (distancing adj2 (intervention? or measure?)).ti,ab,kf.
23. or/19-22
24. 14 or 18 or 23
25. limit 24 to COVID-19
26. Health Knowledge, Attitudes, Practice/ or health behavior/ or health risk behaviors/ or exp Guideline Adherence/
27. (&quot;adhere to&quot; or adherence or attitude* or barriers or challeng* or compliance or comply*or facilitat* or influenc* or knowledge or perception* or practice*).ti,ab,kf.
28. 26 or 27
29. 25 and 28
30. exp animals/ not humans.sh.
31. 29 not 30
32. (comment or editorial or newspaper article).pt.
33. 31 not 32
34. limit 33 to english language
35. limit 34 to &quot;qualitative (maximises specificity)&quot;
